# HIV Viral Suppression With Unintentional Lenacapavir Monotherapy in Adherence-Challenged Patients: A Case Series

**DOI:** 10.1093/ofid/ofaf330

**Published:** 2025-06-11

**Authors:** Nicky J Mehtani, Janet Grochowski, Anthonia Chimezie, Alix Strough, Sarah Strieff, Monica Gandhi

**Affiliations:** Division of General Internal Medicine, University of California, San Francisco, San Francisco, California, USA; Whole Person Integrated Care, San Francisco Department of Public Health, San Francisco, California, USA; Division of HIV, Infectious Diseases and Global Medicine, University of California, San Francisco, San Francisco, California, USA; Division of HIV, Infectious Diseases and Global Medicine, University of California, San Francisco, San Francisco, California, USA; Whole Person Integrated Care, San Francisco Department of Public Health, San Francisco, California, USA; Whole Person Integrated Care, San Francisco Department of Public Health, San Francisco, California, USA; Division of HIV, Infectious Diseases and Global Medicine, University of California, San Francisco, San Francisco, California, USA

**Keywords:** long-acting antiretroviral therapy, people who use drugs, vulnerable populations

## Abstract

Lenacapavir, a long-acting human immunodeficiency virus (HIV) capsid inhibitor, is thought to have a low resistance barrier based on current, limited data. We describe 4 patients experiencing homelessness with severe mental illness who maintained HIV suppression (<30 copies/mL) despite unintentional receipt of lenacapavir monotherapy for 9–38 weeks, highlighting its potential among adherence-challenged populations.

Maintaining adherence to daily oral antiretroviral therapy (ART) is particularly challenging for unstably housed people with human immunodeficiency virus (HIV) [1]. Structural and psychosocial barriers—including inconsistent healthcare access, lack of safe medication storage, and co-occurring mental health and substance use disorders (SUD)—frequently contribute to suboptimal adherence, virologic failure, and increased AIDS-associated morbidity [1]. Long-acting injectable ART (LA-ART) medications, including intramuscular cabotegravir/rilpivirine (CAB/RPV) and subcutaneous lenacapavir (LEN)—an HIV-1 capsid inhibitor administered every 26 weeks—have emerged as promising alternatives for individuals struggling with adherence [2–4]. Based on data from our groups and others [2–6], United States (US) guidelines now recommend CAB/RPV for viremic people with HIV (PWH) experiencing oral ART adherence challenges on a case-by-case basis [7, 8].

LEN is currently approved for heavily treatment-experienced PWH in combination with an optimized background regimen (OBR) [9]. A case series of 34 patients receiving LEN with CAB (dosed every 4–8 weeks) reported high virologic suppression (94%) over a median of 8 weeks [4]. However, asynchronous dosing of LA-ART agents may increase risks of missed doses and genotypic resistance, particularly for LEN, which has been associated with a low resistance barrier when functional monotherapy occurs due to nonadherence or underlying resistance to OBR components [10].

We describe 4 unstably housed PWH with severe mental illness and SUD who unexpectedly maintained viral suppression despite prolonged, unintentional periods of LEN monotherapy. These cases highlight LEN's potential for populations facing adherence challenges and raise critical questions about resistance risk in real-world settings.

## METHODS

Patients with prolonged LEN monotherapy (≥4 weeks) were identified by providers leading LA-ART implementation at 2 San Francisco clinics serving vulnerable PWH: Ward 86 at the University of California, San Francisco, an academic HIV clinic that provides care for ≥2600 PWH annually, and the Maria X. Martinez Health Resource Center (MXM), a community clinic providing transitional care for ≥4000 homeless individuals, including ≥100 PWH. Between June 2021 and February 2025, Ward 86 initiated 431 patients on LA-ART, including 46 on LEN, while MXM initiated 25, including 7 on LEN.

Patients in this case series were prescribed LEN with other LA-ART agents due to adherence challenges with daily oral medications and baseline resistance to CAB, RPV, or both. We included individuals who experienced LEN monotherapy for ≥4 weeks due to missed or delayed concurrent LA-ART dosing. On-time CAB/RPV injections were defined as those given within 28 or 56 ± 7 days after the prior dose for monthly or bimonthly schedules, respectively, while those given >7 days late were considered delayed and associated with LEN monotherapy. Adherence to LEN was similarly categorized as on-time (26 ± 2 weeks following the prior dose) or delayed (>28 weeks), with delayed injections considered likely to result in subtherapeutic LEN levels.

Data abstracted from electronic medical records included patient demographics (age, sex, race/ethnicity, sexual orientation, housing status), mental health and SUD comorbidities, LA-ART components, injection dates, HIV RNA loads, CD4 cell counts, nonnucleoside reverse transcriptase inhibitor (NNRTI) and integrase strand transfer inhibitor (INSTI) resistance mutations, and durations of LEN monotherapy.

The authors are directly involved in the clinical care of included patients. Retrospective reporting of de-identified data was conducted under existing institutional review board approvals for clinical quality improvement.

## RESULTS

Four patients experiencing ≥4 weeks of unintentional LEN monotherapy were identified between October 2023 and December 2024, 2 from each clinic. Collectively, these individuals experienced 8 episodes of LEN monotherapy, ranging from 4 to 38 weeks (Table 1). Despite these extended periods without concurrent LA-ART dosing, each maintained HIV viral suppression (<30 copies/mL). In addition to housing instability, all had severe SUD and psychotic-spectrum conditions. Three were of non-White race/ethnicity (2 Black, 1 Hispanic), 3 were men who have sex with men, and all were ART experienced. LEN was co-prescribed with CAB/RPV or RPV due to documented or suspected baseline resistance to NNRTIs, INSTIs, or both.

**Table 1.** Case Series of Patients With Periods of Prolonged (≥4 Weeks), Unintentional Lenacapavir Monotherapy

Patient ID	Housing Status and Mental Health Comorbidities	LA-ART Regimen	NNRTI Mutations	INSTI Mutations	LEN Monotherapy Duration^a^	HIV RNA, copies/mL		CD4, Cells/μL	
						Before LEN	Post-LEN Monotherapy	Before LEN	Post-LEN Monotherapy
A	Street/sheltered homelessness Methamphetamine use disorder PTSD, major depression, substance-induced psychosis	SQ LEN q26w + IM CAB/RPV q4w	K101P, K103N, V108I, E138K, Y181C	E138K, G140S, Q148R	38 weeks	177 992	<30	187	385
B	Sheltered homelessness Methamphetamine use disorder PTSD, bipolar disorder	SQ LEN q26w + IM CAB/RPV q4w	K101E, Y181C	…	10 weeks	<30	<30	391	427
C	Street/sheltered homelessness Methamphetamine use disorder Substance-induced psychosis	SQ LEN q26w + IM RPV q8w		G140, Q148H	18 weeks	16 597	<30	22	NA
					12 weeks	<30	<30	…	92
D	Unstably housed/couch-surfing Opioid use disorder Major depression, psychosis NOS	SQ LEN q26w + IM CAB/RPV q4-8w	V90I, K103N, L283I	T66I	6 weeks	<30	<30	243	NA
					9 weeks	<30	<30	…	197
					4 weeks	<30	<30	…	312
					8 weeks	<30	<30	…	NA

Abbreviations: CAB/RPV, cabotegravir/rilpivirine; HIV, human immunodeficiency virus; IM, intramuscular; INSTI, integrase strand transfer inhibitor; LA-ART, long-acting antiretroviral therapy; LEN, lenacapavir; NA, not available; NNRTI, nonnucleoside reverse transcriptase inhibitor; NOS, not otherwise specified; PTSD, posttraumatic stress disorder; q4w, every 4 weeks; q4–8w, every 4 to 8 weeks; q8w, every 8 weeks; q26w, every 26 weeks; SQ, subcutaneous.

^a^Durations of LEN monotherapy refer to periods during which concentrations of concurrently prescribed LA-ART agents were presumed to have been subtherapeutic due to delayed or missed dosing. These do not include additional periods of functional LEN monotherapy due to baseline resistance to concurrently prescribed CAB and/or RPV.

### Case Summaries

Patient A was initially prescribed CAB/RPV alone, but LEN was added when he developed viremia following 2 late CAB/RPV injections (delayed by 12 and 21 days). An HIV RNA genotype subsequently revealed extensive new CAB and RPV resistance. CAB/RPV was discontinued, and he was advised to initiate daily oral ART, which was declined. Thirty-eight weeks after his single LEN injection, he was found to be virally suppressed, despite an estimated 10 weeks of presumed subtherapeutic LEN monotherapy. This unexpected outcome led to reinitiation of LEN + CAB/RPV, with the latter injectables added back despite high-level resistance to optimize virologic containment and care engagement in the setting of limited options and the patient's continued refusal of oral ART. He has remained suppressed on this regimen for an additional ≥40 weeks.

Patient B received LEN + CAB/RPV for over a year prior to experiencing a disruption in healthcare due to unplanned travel, resulting in a 10-week delay in CAB/RPV injections. This disruption began 20 weeks after his last LEN injection, ultimately resulting in 2 weeks of suspected subtherapeutic LEN prior to returning to care and reinitiating both injections, at which time he was found to have maintained virologic suppression.

Patient C initiated LEN + RPV during a hospitalization due to baseline INSTI resistance, long-standing challenges with oral ART, and severe immunosuppression (CD4 count, 22 cells/μL). While follow-up was expected to be difficult, she achieved and maintained viral suppression on this regimen, despite 2 episodes of LEN monotherapy lasting 18 and 12 weeks, respectively.

Patient D received most of his care in emergency department and hospital settings due to unstable housing. After 6 months on LEN + CAB/RPV, he experienced 4 episodes of delayed CAB/RPV dosing, resulting in LEN monotherapy periods lasting anywhere from 4 to 9 weeks.

## DISCUSSION

While clinical trial data suggest that LEN has a low genetic barrier to resistance when used as functional monotherapy [10], this case series provides unexpected real-world observations of sustained HIV viral suppression despite up to 38 weeks of LEN monotherapy. Given that all patients faced extensive structural and psychosocial barriers to oral medication adherence—including severe mental illness, SUD, and homelessness—these cases raise important questions about LEN's resistance profile, pharmacologic properties of HIV capsid inhibitors, and the potential of LEN in combination ART to support adherence-challenged populations. Importantly, these observations must be interpreted with caution, as LEN monotherapy has been linked to virologic failure and resistance in controlled studies [9, 10].

It is worth noting that, to date, beyond the 4 cases outlined here, all 53 patients from Ward 86 and MXM initiated on LEN in combination ART have maintained HIV viral suppression to <200 copies/mL at the latest follow-up, despite several having imperfect adherence to the OBR (ie, <4 weeks of functional monotherapy). This is significant given that CAPELLA (NCT04150068), the clinical trial leading to LEN's US Food and Drug Administration approval, included only 72 participants [9], underscoring the importance of further data to inform LEN treatment. In CAPELLA, 14 cases of virologic failure with LEN resistance emerged by 104 weeks, 10 of which were associated with OBR nonadherence and 4 with underlying resistance to all OBR components [10 ]. However, the trial did not assess OBR adherence in participants who maintained virologic suppression, leaving open the possibility that others also experienced prolonged periods of OBR nonadherence, similar to the cases presented here.

The mechanisms underlying sustained virologic suppression observed in these cases remain unclear. Importantly, of the 14 LEN resistance cases in CAPELLA, 7 resuppressed on LEN, either with adherence counseling or switching the OBR [10]. In addition, M66I, a common LEN-associated resistance mutation, confers an HIV replication capacity of 1.5% compared to wild-type [10]. Therefore, while persistent viral suppression in our cases precluded genotypic resistance analyses, it is possible that LEN mutations evolved but resulted in poor viral fitness. Alternatively, these findings could suggest that pharmacokinetic factors, indirect immune responses, or other host factors contribute to viral suppression in the context of LEN monotherapy.

While LEN monotherapy should never be endorsed as a treatment strategy, these cases raise important considerations for clinical decision-making in settings where healthcare disruptions are common and/or unavoidable. People who use drugs and those experiencing homelessness account for an increasing proportion of PWH in the US and face structural challenges in adhering to oral medications [1], yet providers remain hesitant to initiate intramuscular CAB/RPV due to concerns for treatment lapses. Several studies in real-world settings have documented cases of emergent NNRTI and INSTI resistance on CAB/RPV, even with minimally delayed dosing [3, 6, 11], and pharmacokinetic data from the Swiss HIV Cohort Study demonstrated substantially lower cabotegravir concentrations than those previously reported in clinical trials [12]. The unexpected, sustained viral suppression observed in the cases presented here thus suggests that LEN may serve as a critical component of LA-ART regimens for patients at risk of care disengagement. Given the substantial improvements in CD4 cell counts, immune function, and mortality associated with LA-ART in adherence-challenged populations, LEN-containing regimens may provide more benefits than risks, particularly for those with low CD4 cell counts.

Another key question is whether, when co-prescribed with CAB/RPV, the longer pharmacokinetic tail of LEN could offer relative protection against NNRTI and INSTI resistance in patients prone to adherence gaps. For example, patient B's experience suggests that LEN may have prevented further NNRTI and INSTI resistance despite a 10-week delay in CAB/RPV dosing. This raises the possibility of incorporating LEN into asynchronous LA-ART regimens with CAB/RPV, even for patients without baseline NNRTI or INSTI resistance.

Ultimately, while monotherapy is not a viable long-term HIV treatment approach, this care series offers valuable real-world insights into how LEN may function as a temporary bridge or harm reduction tool for highly vulnerable populations. It underscores the importance of individualized care strategies, particularly in nontraditional contexts. Further studies, including large observational cohorts, clinical trials, and a national registry of LEN cases, would provide a more comprehensive understanding of LEN's resistance profile and its potential role in HIV treatment for those facing significant adherence barriers.

## Notes

***Author contributions.*** N. J. M. and M. G. conceived this case series. J. G., A. C., A. S., and S. S. contributed to data collection and patient outreach. All authors contributed to data interpretation. N. J. M. wrote the initial draft, and all authors contributed to editing the final manuscript.

***Patient consent.*** Retrospective reporting of de-identified patient data was conducted under existing institutional review board approvals for clinical quality improvement, which did not necessitate written consent.

***Financial support.*** The study was supported by the National Center for Advancing Translational Sciences (grant number 2KL2TR001870 to N. J. M.) and the National Institute of Allergy and Infectious Diseases (grant number 5R37AI098472 to M. G.).
